# Comparison of ImmunoCAP and Immulite serum specific IgE assays for the assessment of egg allergy

**DOI:** 10.1186/s13223-016-0134-0

**Published:** 2016-06-10

**Authors:** François Graham, Philippe Bégin, Louis Paradis, Jonathan Lacombe-Barrios, Jean Paradis, Anne Des Roches

**Affiliations:** Allergy and Immunology, Centre Hospitalier de l’Université de Montréal Hôpital Notre-Dame, Montreal, QC Canada; Allergy and Immunology, Centre Hospitalier Universitaire Sainte-Justine, 3175 Chemin de la Cote Sainte-Catherine, Montréal, QC H3T1C5 Canada

**Keywords:** Egg allergy, Food allergy, Immulite, ImmunoCAP, UniCAP, Specific IgE assays

## Abstract

Egg specific IgE levels are frequently used in combination with skin-prick tests to guide clinical decisions and to monitor egg allergy evolution in children. We compared both Immulite and ImmunoCAP egg specific IgE assays in egg allergic children, and found a linear correlation between both assays with a mean Immulite:ImmunoCAP ratio of 3. This is relevant information for clinicans wishing to estimate values from one assay to the other, as most literature has been published using the ImmunoCAP system.

## Findings

Egg allergy represents one of the most common food allergies encountered in pediatric practice, with an estimated prevalence of 0.5–2 % in infants and young children [1]. Many egg allergic children are able to tolerate baked eggs [2], which can greatly improve quality of life. Egg specific IgE levels are frequently used in combination with skin-prick tests (SPT) to guide clinical decisions and to monitor egg allergy evolution in children. Most reports on egg allergy have been using the ImmunoCAP (Phadia AB, Uppsala, Sweden) assay [3–5], which is a problem for the fraction of clinicians who do not have access to it, as their lab works with Immulite (Siemens Healthcare Diagnostics, Tarrytown, New York) for technical or administrative reasons. In this context, clinical literature is challenging to interpret and implement in practice. Some would recommend simply not using this ImmunoCAP literature, but this would mean depriving patients from useful information to guide management and therapy. Interestingly, it has been suggested that although egg-specific IgE results from either assays cannot be substituted [6], they may be adapted so that the results may still be used to guide management [7, 8].

The objective of this study was to directly compare Immulite and ImmunoCAP egg white-specific IgE assays and to determine whether their measurements can be applied equivalently and/or adapted to guide clinical management of egg allergic children.

Briefly, 37 egg allergic patients between 2 and 13 years of age were enrolled at Sainte-Justine University Hospital Center (Montreal, Canada) from July 2013 to January 2014. Patients with egg allergy had either a positive OFC or a history of at least one sign or symptom of allergy (ocular, respiratory, gastrointestinal, or cardiovascular) occurring within 1 h of egg ingestion and persistent sensitization at time of evaluation confirmed by a positive egg white skin prick test (3 mm greater than control), and either ImmunoCAP specific IgE levels ≥0.35 kU/L or Immulite specific IgE levels ≥0.1 kU/L. The project was approved by the ethics committee of Sainte-Justine University Hospital Center.

Patients’ serum was aliquoted into two separate samples and sent on dry ice for analysis at the laboratories of the University of Montreal Hospital Center (CHUM) and Sainte-Justine University Hospital Center, each using a different specific IgE assay system: ImmunoCAP Phadia 250 and Siemens DPC Immulite 2000.

Descriptive analysis consisted of medians and range. Immulite and ImmunoCAP values were compared using Pearson’s correlation (GraphPad Prism 6, San Diego, CA).

The median age of patients was 6.5 years (range, 2–13) and the median age at first reaction to eggs was 12 months (range, 4–96). The age of worst reaction was a median of 5.5 years before testing. Eighteen patients (49 %) had a history of anaphylactic reactions to eggs and 10 (27 %) tolerated baked eggs, while most of the remainder had never ingested baked egg before. Median egg white skin prick test diameter at time of specific IgE measurement was 10 mm (range, 3–25 mm).

In the whole cohort, Immulite median egg white-specific IgE levels was 24.80 [range, 0.72–100] kU/L compared to 6.45 [range, 0.33–100] kU/L for ImmunoCAP. In the subgroup tolerating baked eggs (n = 10), median egg white-specific IgE levels was 5.2 [range, 1.13–28.1] kU/L using Immulite and 3.17 [range, 0.38–8.93] kU/L using ImmunoCAP. When examining the subgroup of patients with anaphylactic reactions to eggs (n = 18), the median egg white-specific IgE levels using Immulite was 17.4 [range, 0.715–100] kU/L compared to 5.90 [range, 0.33–100] kU/L for ImmunoCAP.

**Fig. 1:**
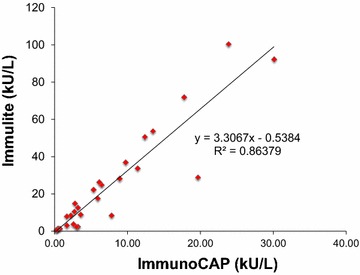
Correlation plot of egg white specific IgE levels as measured with ImmunoCAP^®^ and Immulite^®^

Thus, as previously suggested, Immulite and ImmunoCAP egg-specific IgE values could not be substituted [6]. However, values were highly correlated (Pearson correlation factor of 0.864; Fig. 1) with egg white-specific IgE levels measured by Immulite a mean of 3.02 (± 0.44) times higher than when measured by ImmunoCAP(n = 29, ImmunoCAP values ≤30 kU/L). This correlation ratio was lost with ImmunoCAP values higher than 30 kU/L, which likely reflects the fact that values higher than 100 kU/L for Immulite exceeded the top point of the calibration curve. One can expect that diluting the samples with an appropriate serum diluent and reanalyzing the samples would have preserved this linear correlation [8]. Interestingly, the ImmunoCAP:Immulite ratio was of 1.64 in the subgroup tolerating baked eggs (n = 10). The significance of this lower ratio is difficult to interpret due to the small n in this sub-group. A possibility is that individual allergen components (i.e., ovomucoid vs ovalbumin) may be measured differently in each assay, which would become apparent when comparing subgroups with different sensitivity profiles (baked good tolerant vs allergic).

These observations are in line with previous studies. Wang et al. found an Immulite:ImmunoCAP ratio of 3.7 for egg-white specific IgE in 50 atopic patients [6]. Although the correlation coefficient was not included, qualitatively it appeared to be very high. Another study from South Korea evaluated atopic patients 1–75 years of age and found a very similar Pearson’s correlation coefficient of 0.845 for egg white-specific IgE when comparing both assays [7]. Hamilton et al. [8] also found a mean Immulite:ImmunoCAP ratio of 4.85 and a high coefficient of determination of 0.95 in children aged 1–16 with a history of egg allergy (no skin prick tests or challenge), which is comparable to our results.

In conclusion, because of variability between Immulite and ImmunoCAP specific IgE assays, it is preferable to use a single assay to monitor the evolution of egg allergy and to assess the development of tolerance. This said, a linear correlation does exist between both assays, as has been observed in four independent cohorts including ours. Therefore in the absence of access to ImmunoCAP, a factor of 3–5 could be applied to egg-specific IgE published thresholds to guide clinical decisions. Although imperfect, this approach remains in our opinion preferable to withholding useful clinical information from patients and clinicians.

## Abbreviations

SPT: skin-prick test
OFC: oral food challenge
